# Let's look at leeks! Picture books increase toddlers' willingness to look at, taste and consume unfamiliar vegetables

**DOI:** 10.3389/fpsyg.2014.00191

**Published:** 2014-03-11

**Authors:** Philippa Heath, Carmel Houston-Price, Orla B. Kennedy

**Affiliations:** ^1^School of Psychology and Clinical Language Sciences, University of ReadingReading, UK; ^2^Department of Food and Nutritional Sciences, University of ReadingReading, UK

**Keywords:** exposure, picture books, fruit and vegetables, visual preference, willingness to taste, consumption

## Abstract

Repeatedly looking at picture books about fruits and vegetables with parents enhances young children's visual preferences toward the foods in the book (Houston-Price et al., 2009a) and influences their willingness to taste these foods (Houston-Price et al., 2009b). This article explores whether the effects of picture book exposure are affected by infants' initial familiarity with and liking for the foods presented. In two experiments parents of 19- to 26-month-old toddlers were asked to read a picture book about a liked, disliked or unfamiliar fruit or vegetable with their child every day for 2 weeks. The impact of the intervention on both infants' visual preferences and their eating behavior was determined by the initial status of the target food, with the strongest effects for foods that were initially unfamiliar. Most strikingly, toddlers consumed more of the unfamiliar vegetable they had seen in their picture book than of a matched control vegetable. Results confirm the potential for picture books to play a positive role in encouraging healthy eating in young children.

## Introduction

Picture books provide a rich, indirect source of information about the world with which children can supplement the knowledge they acquire through personal experience. It is therefore of interest to ascertain what types of information children acquire from picture books, and under what circumstances children spontaneously transfer what they have learned from pictures to the real world. Previous research has established, for example, that 24-month-old infants generalize words that have been taught as labels for pictures to the objects themselves (Preissler and Carey, 2004; Ganea et al., 2008), while older preschoolers transfer information they have learned about the biological properties of animals in picture books to the animals themselves (Ganea et al., 2011). Such research has established a number of factors that influence the success with which children transfer their learning from pictures to the real world. First, the child must understand the symbolic relationship between pictures and the objects and events these represent; such an awareness of how pictures represent reality is first evident during the second year of life (Ganea et al., 2009). Second, transfer of learning is best supported by pictures that closely resemble their real-world referents; thus, photographic images facilitate generalization of learning relative to less realistic images (Ganea et al., 2008). Third, generalization of learning is more likely to occur in similar contexts to those in which the learning occurred. In a study by Simcock and Dooley (2007), for example, infants showed lower levels of imitation of an action sequence toward an object when they moved to a different test room between seeing the action in a picture book and being presented with the object themselves.

Previous research by our group has built on this work to investigate whether looking at picture books about healthy foods affects preschoolers' behavior toward the foods depicted. Houston-Price et al. (2009a) provided the parents of 17- to 27-month-old toddlers with books containing photographs and information about fruits and vegetables and asked them to read these with their children on a daily basis for 1, 2, or 3 weeks. In this study, the impact of the books was measured in terms of children's visual preferences for the exposed foods. When children were shown pairs of pictures of foods, such that one food in each pair had been included in the child's book and one had not, they spent significantly longer looking at the fruits and vegetables they had seen in their books. The largest impact on looking time was shown by children whose parents had been asked to read the book with them every day for a fortnight (who actually recorded completing an average of nine readings). Importantly, a looking preference for the exposed foods was evident both when the pictures presented in the visual preference test were identical to those children had seen in their books and when new pictures of the exposed foods were used at test. This led Houston-Price et al. (2009a) to argue that children's longer looking times toward the target foods in this study were not solely driven by the perceptual familiarity of the exposed pictures, but rather reflected children's interest in the foods themselves.

To test this hypothesis, Houston-Price et al. went on to explore whether looking at picture books about fruits and vegetables influences children's willingness to taste these foods, as well as their interest in looking at them (Houston-Price et al., 2009b). Parents were asked to read a picture book to their 21- to 24-month-old children every day for a fortnight. The books featured two familiar foods (e.g., sweetcorn and strawberries) and two unfamiliar foods (e.g., radishes and lychees). After the reading period, children took part in a taste test, in which they were offered a plate of four vegetables, followed by a plate of four fruits. Each child had seen pictures of two of the four items on each plate in their books. The children were encouraged to taste all of the foods presented and the order in which foods were tasted was recorded. Children tasted significantly more of the foods that were expected to be familiar to them, displaying a neophobic pattern of behavior that is typical of this age group (Cashdan, 1994; Raudenbush and Frank, 1999; Cooke et al., 2003). However, the order in which children approached the foods that were not expected to be familiar was affected by the book they had seen. Children tasted the unfamiliar fruit they had seen in their book before the unfamiliar fruit they had not seen in their book. This study therefore provides preliminary evidence that picture books can influence the foods that children are willing to taste.

In our view, these findings are worth pursuing, as they suggest that a positive attitude toward healthy foods might be engendered in children before they have even tasted them. This is important because, without prior visual exposure, children need to taste a new food between 8 and 15 times before they will accept it into their diet (Birch and Marlin, 1982; Birch et al., 1987; Sullivan and Birch, 1990; Wardle et al., 2003a,b; Lakkakula et al., 2010). It is often difficult for parents to provide this number of exposures, given the challenging behavior they are confronted with when they ask their toddler to try a new food. In fact, parents typically offer a new food to their child on only three to five occasions before giving up (Carruth and Skinner, 2000; Carruth et al., 2004). As a result, children not only fail to receive sufficient exposures to new fruit and vegetables for these to become accepted into their diet, but parents also tend to fall back on foods that are known to be liked by the child, reinforcing the child's desire for these (Nicklaus, 2011). If children's willingness to taste a new food is enhanced by a period of picture-book exposure prior to introducing the food at mealtimes, parents' efforts to provide their children with a varied and healthy diet might perhaps be facilitated.

However, while picture books were found to have a positive effect on children's willingness to taste the unfamiliar fruits in Houston-Price et al.'s (2009b) study, it is important to note the unexpected negative result reported in the same study. Although children were more willing to taste the foods that were expected to be familiar to them, the vegetables that fell into this category (carrots and sweetcorn) were *less* likely to be tasted if children had seen these in their book. That is, while looking at pictures of lychees for 2 weeks increased children's willingness to taste these in a subsquent taste test, looking at pictures of carrots for the same period had the opposite effect. Work is therefore needed to establish the types of foods for which picture-book exposure has the desired effect. It is interesting to note that a similar decrease in desire for a food is sometimes found to follow repeated taste exposures. For example, when Liem and Zandstra (2009) asked 12-year-old children to consume the same unfamiliar snack food every day for 3 weeks, children's desire for the snack declined over time due to the monotony of eating the same food every day. Even after a single lunch session, preschoolers and adults may display “sensory specific satiety,” a decrease in the reported pleasantness of the recently consumed flavor or texture (Birch and Deysher, 1986; Rolls, 1986). One account of these findings proposes that over-exposure to a food devalues its worth (Brondel et al., 2007). Brondel et al. (2007) asked 144 adults to evaluate the pleasantness of six different foods before and after they were invited to consume these foods “*ad libitum*.” Individuals consumed greater quantities of the foods rated as having higher hedonic value but the pleasantness ratings given to these foods decreased following consumption relative to uneaten foods. Thus, although individuals choose to eat the foods that they rate highly in terms of hedonic liking, their desire for these foods lessens as they gain exposure to them. While no measure was taken of the extent to which children liked the familiar foods in Houston-Price et al.'s (2009b) study, it is possible that children's disinterest in tasting the exposed familiar vegetables might have a similar cause; as with repeated taste exposure, repeated visual exposure to foods that are already liked and/or familiar might decrease a child's interest in consuming them. The aim of the studies reported here was therefore to examine whether a child's prior familiarity with or liking of a fruit or vegetable, as reported by parents, moderates the extent to which picture-book exposure affects the child's willingness to look at and taste the foods depicted.

We report two experiments, in each of which a picture book about a liked, disliked or unfamiliar fruit or vegetable was repeatedly read to 18- to 24-month-old children by their parents. Experiment 1 examined the impact of the books on children's visual preferences for exposed (“target”) foods vs. non-exposed (“control”) foods. As in Houston-Price et al. (2009a), we compared looking times toward both seen pictures and new pictures of the target foods. Experiment 2 investigated how a food's initial status impacts on the books' effectiveness as a means of increasing children's willingness to taste target foods. Based on the findings of Houston-Price et al. (2009b), we hypothesized that, in both studies, unfamiliar foods would be subject to stronger exposure effects than familiar (liked and disliked) foods, and that the intervention would be least effective for foods that were already liked.

## Experiment 1

### Method

#### Participants

One hundred and fifty-four toddlers aged between 19 and 26 months, all reported to have normal hearing and vision, and their parents were recruited from the University of Reading's Child Development Group database of families who had expressed an interest in taking part in research. Of these, 22 children were excluded for failing to meet criteria for participation (as detailed in the Procedure) and 13 families withdrew from the visual preference test due to ill health or other commitments. The final sample consisted of 119 children (60 males and 59 females) with a mean age of 21 months 26 days (range 19 months 24 days to 26 months 15 days). Demographic information (provided by more than 90% of those who completed the study) indicated that 87% of participating families were white and 76% included at least one parent educated to graduate level.

#### Materials

Previously-collected ratings of toddlers' familiarity with and liking of 39 fruits and 48 vegetables on a “Food Familiarity and Liking Questionnaire” (FFLQ) were used to select the food items for this study. These ratings were provided by the parents of 93 children (57 boys and 36 girls) aged between 16 and 24 months, also recruited from the University of Reading's Child Development Group database, between 2006 and 2009. The six vegetables identified as the most liked, the most disliked and the most unfamiliar to children according to these ratings were selected as stimuli for the corresponding initial status conditions of the current study (see Supplementary Material). Similarly, the six most liked fruit and the six most unfamiliar fruit were selected for the corresponding fruit categories. As it was not possible to identify six fruits that were commonly disliked by children at this age, there was no “disliked fruit” condition in this study.

An individual picture book was produced for each of the fruits and vegetables in each initial status set (an example of a book can be seen in Supplementary Material). The layout and format of each book was identical. Books were A5 in size and constructed of strong card; they were brightly colored and written in a style suitable for 18- to 24-month-old children. Books consisted of seven pages of pictures and information about the target fruit or vegetable. The first page of each book provided instructions to parents about how to read the book and the last page contained a tick-sheet reading record upon which parents were asked to note how many times they looked at the book with their child. On each of the remaining five pages a large photograph and information about the chosen fruit or vegetable was displayed. Photographs were matched for type across books and recounted the progression of the fruit or vegetable from “farm to fork” (i.e., from what the food looks like when growing in the field to its appearance when presented for eating). The supporting sentences described the pictures and provided additional information about the food shown (see Supplementary Material).

Visual preference testing took place in a three-sided visual preference booth with a large back-projection screen measuring 1.5 × 0.6 m on the rear wall. A chair for parents to sit on, while holding the child on their lap, was placed one meter away from the screen. Adobe Photoshop 4.0 was used to generate 10 different 320 × 200 pixel, 256-color images of each fruit or vegetable against a white background. Five of the images of each food were identical to those displayed in the picture books, while five were new but easily-recognizable pictures of the food (subsequently referred to as “seen pictures” and “new pictures” respectively). Images were displayed side by side on the screen at infant eye height; images measured 24 × 16 cm and were separated by a gap of 25 cm. A 24 × 40 cm image of a popular character from a children's television programme was used to refocus children's attention to the center of the screen between trials. Two auditory tokens of the word “Look” were recorded by an adult female voice in infant-directed speech, one to be used during experimental trials, the other to be used between trials to attract infants' attention to the screen. The booth had low-level lighting so that infants' looking direction could be captured by three infrared cameras situated immediately above the two image locations and central point of the screen.

#### Procedure

Parents were contacted by telephone and given a brief description of the experiment. If they gave consent for their child to participate, the child was immediately randomly assigned to one of five initial status groups: Liked Vegetable (*N* = 24), Disliked Vegetable (*N* = 23), Unfamiliar Vegetable (*N* = 24), Liked fruit (*N* = 24), and Unfamiliar Fruit (*N* = 24). During the initial telephone call, the researcher read out the list of six foods in the initial status category to which the child had been assigned and asked the parent whether the child liked, disliked or had not tried each food. If parents reported that the child's familiarity with or liking of two or more of the six foods matched the expected status of the food, one of these was randomly selected to be the target (exposed) food and another was randomly selected to be the control (non-exposed) food for that child. If fewer than two foods matched the status of the child's allocated group, the child was not included in the study. An appointment was made for parents and children to take part in a visual preference test at the School of Psychology and Clinical Language Sciences at the University of Reading a few weeks later.

Parents were then sent a picture book about the child's target food in the post, with instructions to read the book with their child for approximately 5 min a day for 14 days. Parents were invited to use the words provided and their own words when reading the book and were asked to complete the tick sheet each time they read it. If, after receiving the book, a parent asked to rearrange their appointment for the visual preference test, they were asked to stop reading the book and to resume reading it nearer to the visit date, ensuring that the child received a total of 14 exposures. On three occasions, the child had already seen the book 14 times before their visit was rescheduled; in these cases, parents were asked to take a break from looking at the book and to resume reading it again 3 days before their visit.

Parents visited the University on weekday mornings or afternoons at times they found convenient and when the child was alert but not hungry. On arrival, the experimenter spoke with the parent and played with the child for a few minutes so that they felt relaxed in the laboratory environment. Parents were then invited to sit with their child on their lap in the visual preference booth for the preference test. Parents were asked to keep their eyes closed throughout the test period, to ensure they did not influence their child's behavior. The child was shown a series of trials, each lasting 7 s. On each trial, a pair of pictures was shown side-by-side on screen; one was a picture of the target food that the child had been exposed to in their picture book, the other was a picture of the control food selected from the same initial status set. Picture pairs were matched for type, such that both showed a food growing on the plant, for example. Children were quasi-randomly assigned to take part in a “seen” or “new” pictures condition. The first 60 participants recruited took part in the “seen pictures” condition, and saw the same five pictures of the target food in their preference test that they had seen in their picture books. Remaining participants (*N* = 59) took part in a “new pictures” condition, and saw five new pictures of their target food during their preference test. The five pairs of pictures were displayed twice, once with the target food on the left side of the screen and once with the target food on the right side of the screen, making 10 trials in total. Picture pairs were shown in a random order. One hundred milliseconds after the onset of each trial, the audio instruction “*Look!*” was played from speakers situated above and below the screen to direct children's attention to the screen. The researcher controlled the start of each trial and, when necessary, played a second instruction to “*Look!*” between trials to attract the child's attention toward the screen.

#### Coding and measures

Video recordings of children's fixation on each image during each trial were coded off-line on a frame-by-frame basis using Observer 5.0 Software (http://www.noldus.com/human-behaviorresearch/products/theobserver-xt). Each frame was coded as a look to the left image, right image, between images or away from the screen. Coders were blind to the condition of the child they were coding and to the side of the screen on which the target food was displayed. One researcher coded the full set of recordings and, to check coding reliability, a random sample of 30% of recordings (*N* = 42) was independently coded by a second coder. The mean Cohen's Kappa for concordance between the two scorers' codes for each frame of these recordings was 0.92 (range = 0.78–1.00).

The measure of visual preference used was the “total looking time difference,” the mean difference in the time children spent looking at the target picture and the control picture on each trial, averaged over the 10 test trials and across participants. Thus, mean values greater than zero indicate that children spent more time looking at the target food than the control food. To test the data's suitability for parametric analyses, we examined the standardized residuals for the overall 2 × 3 × 2 ANOVA (food type x initial status set x seen/new pictures), which confirmed that the error terms for the total looking time difference measure followed a pattern of normal distribution [Shapiro-Wilk's *W*_(119)_ = 0.99, *p* = 0.50].

### Results and discussion

According to the reading records provided by parents, there was a large range in the number of readings provided. Although books were read an average of 14.03 times (*SD* = 6.20), the smallest number of exposures was 6, while two children saw their book between 40 and 50 times. As there was no correlation between the number of exposures provided and the measure of total looking time difference, *r*_(118)_ = −0.02, *p* = 0.80, and as the two children with the highest number of exposures were not outliers on this measure, we did not exclude any participants on the basis of the number of readings experienced.

Mean looking times toward the target and control foods and mean total looking time differences for the children in each condition can be seen in Table 1.

**Table 1:** **Mean looking times toward target and control foods and mean total looking time differences (target—control) for the children in each condition of Experiment 1**.

**Condition**	***N***	**Looking time to target food Mean (*SD*)**	**Looking time to control food Mean (*SD*)**	**Total looking time difference Mean (*SD*)**
**SEEN PICTURES**				
Liked fruit	12	3146 (343)	2725 (321)	421 (520)
Liked vegetables	12	3016 (481)	2833 (316)	183 (639)
Disliked vegetables	11	3546 (492)	2631 (477)	915 (881)
Unfamiliar fruit	12	3424 (701)	2361 (447)	1063 (1014)
Unfamiliar vegetables	12	3684 (526)	2299 (443)	1385 (876)
All foods	59	3360 (562)	2569 (444)	791 (893)
**NEW PICTURES**				
Liked fruit	12	3002 (367)	2545 (432)	457 (511)
Liked vegetables	12	2786 (468)	2646 (248)	140 (434)
Disliked vegetables	12	2738 (474)	2616 (625)	122 (723)
Unfamiliar fruit	12	2978 (463)	2511 (437)	467 (678)
Unfamiliar vegetables	12	2853 (624)	2500 (433)	353 (623)
All foods	60	2871 (481)	2563 (439)	308 (602)

We first sought to confirm previous findings that picture-book exposure creates a visual preference for target foods. When total looking time differences were averaged across participants in all conditions (*M* = 548 ms, *SD* = 796), a one sample *t*-test confirmed that children looked longer at target foods than control foods, *t*(118) = 7.51, *p* < 0.001. When the same tests were run for the children in each of the initial status groups separately, a significant looking preference for the target foods was found in all three groups [liked foods: *M* = 300 ms, *SD* = 533 ms, *t*(47) = 3.90, *p* < 0.001; disliked foods: *M* = 501 ms, *SD* = 882 ms, *t*(22) = 2.72 *p* = 0.012; unfamiliar foods: *M* = 817, *SD* = 896 ms, *t*(47) = 6.32 *p* < 0.001]. Similarly, children looked longer at target foods whether they saw the same pictures they had seen in their picture books, *t*(58) = 6.80, *p* < 0.001, or new pictures, *t*(59) = 3.96 *p* < 0.001.These results indicate that, regardless of the initial status of the target food or the type of picture shown in the test phase, the picture books enhanced children's visual attention to the exposed foods relative to the non-exposed foods of the same initial status.

To explore the impact of food type (fruit or vegetable), initial status (liked, disliked, or unfamiliar) and picture type (seen or new) on children's looking behavior, the data were entered into a 2 × 3 × 2 ANOVA. There was no main effect of food type, *F*_(1, 109)_ = 0.36, *p* = 0.55, and no interactions between food type and initial status set, *F*(1, 109) = 1.73, *p* = 0.19, food type and picture type, *F*(1, 109) = 0.79, *p* = 0.38, or food type, initial status set and picture type, *F*(1, 109) = 0.38, *p* = 0.54. The impact of the picture books was equivalent whether children saw pictures of fruit or vegetables.

**Figure 1:**
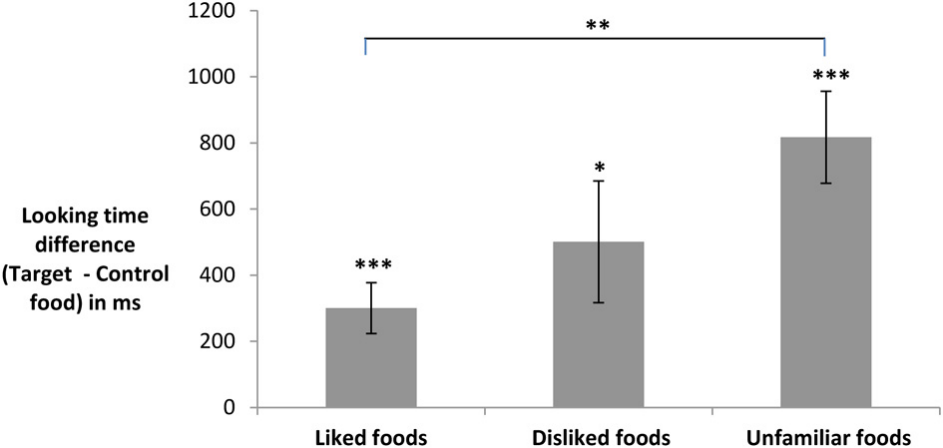
**Mean total looking time differences (in ms) for initially liked, disliked, and unfamiliar foods.** Differences greater than 0 ms indicate that children looked longer at target than control foods. One-sample *t*-tests against chance (0): ^*^p = 0.02, ^***^p < 0. 001. Pairwise comparison: ^**^p = 0.002.

In contrast, the hypothesized influence of children's initial familiarity with or liking for the foods was seen. There was a significant main effect of the initial status of the target food, *F*(2, 109) = 6.35, *p* = 0.002. As shown in Figure 1, children's preference for the target food was strongest for initially unfamiliar foods and weakest for initially liked foods. *Post-hoc* tests showed that total looking time differences differed between the liked and unfamiliar conditions (Scheffe, *p* = 0.002), with no significant differences between the liked and disliked conditions (Scheffe, *p* = 0.54) or the disliked and unfamiliar conditions (Scheffe, *p* = 0.22). When the children in the liked and disliked initial status conditions were combined to form a “familiar food” group, an independent *t*-test revealed that children's total looking time differences toward unfamiliar foods (*M* = 817, *SD* = 896) were significantly larger than those toward familiar foods [*M* = 365, *SD* = 667; *t*(81) = 2.98, *p* = 0.004]. Thus, while picture book exposure enhanced attention toward target foods for children in all conditions, the intervention was more effective when children read about unfamiliar foods than when they read about foods that were already known to them or liked by them.

The ANOVA also tested the impact of presenting seen pictures or new pictures of the target foods at test. There was a significant main effect of picture type, *F*(1, 109) = 13.06, *p* < 0.001; children showed a greater preference for their target food when presented with seen pictures of this food (*M* = 791 ms, *SD* = 894 ms) than when presented with new pictures (*M* = 308, *SD* = 602 ms). There was also a significant interaction between picture type and initial status set, *F*(2, 109) = 4.15, *p* = 0.018; *post-hoc* t-tests established that the larger exposure effect among children who saw seen pictures at test was true for foods that were initially disliked, *t*(21) = 2.37 *p* = 0.028, or unfamiliar, *t*(46) = 3.50 *p* = 0.001, but not for foods that were initially liked, *t*(46) = 0.023 *p* = 0.98. Thus, while the intervention had a positive impact on children's interest in looking at the target food regardless of the pictures presented at test, the effect was stronger for previously seen pictures when foods were initially disliked or unfamiliar.

In summary, the results of Experiment 1 indicate that the impact of picture-book exposure on children's visual preferences varies according to the initial status of the food presented in the book—with the strongest effects for initially unfamiliar foods—and the stimuli used to assess preferences at test. Importantly, in no condition was exposure found to have a negative effect upon children's looking behavior. The finding that exposure effects were greater when the preference test used the same pictures shown in children's picture books suggests that perceptual familiarity was a factor in children's behavior in this study (Zajonc, 1968, 2001; Bornstein and D'Agostino, 1994; Monahan et al., 2000). However, as positive exposure effects were also found for pictures that had not been seen before, the intervention may also have influenced children's interest in the depicted foods themselves. Experiment 2 set out to directly test this possibility by exploring whether looking at a book about a food affects children's behavior toward the food itself. Again, we manipulated the food's initial status in order to elucidate the effects of the book on children's behavior toward initially liked, disliked and unfamiliar foods.

## Experiment 2

Experiment 2 examined children's willingness to taste an initially liked, disliked or unfamiliar vegetable after looking at a picture book about the food for 2 weeks, in comparison to a control food of the same initial status. This study focused solely on vegetables on the grounds that: (a) Experiment 1 found no differences in the effects of the intervention for fruits and vegetables; and (b) as children's least-favored food group, vegetables are a particular challenge for healthy eating interventions (Cashdan, 1998; Skinner et al., 2002; Cooke and Wardle, 2005). After looking at a picture book with parents for 2 weeks, children took part in a laboratory taste test, in which they were offered both the target vegetable they had seen in their book and a non-exposed control vegetable of the same initial status. Willingness to taste the foods was measured in terms of whether a food was tasted, the order in which the two foods were tasted and the encouragement required to persuade the child to taste the food. We also measured the amount of each food consumed. We hypothesized that children would be more willing to taste target foods than control foods, but that the strength of the exposure effect would be moderated by the food's initial status: we expected the strongest effect to be seen for initially unfamiliar foods, and the weakest effect for foods that were already liked.

Experiment 2 additionally examined the accuracy of parents' reports' of children's food likes and dislikes. While parents of children under two are typically present at their children's mealtimes and would be expected to know which foods the child has eaten and their likes and dislikes, previous research has produced mixed results with regard to the accuracy of parental reports. Studies using standard free-recall methods have found that parents overestimate infants' energy and nutrient intake (Baranowski et al., 1991; Fisher et al., 2008), while those using closed-recall methods have found parents to make accurate assessments of their child's fruit and vegetable intake (Linneman et al., 2004). Experiment 2 explored parents' ability to accurately report which vegetables are liked and disliked by their child; we recorded children's eating behavior when they were presented with a pair of foods, one reported to be liked and one reported to be disliked by parents. We hypothesized that children would be more willing to taste the foods that were reported to be liked and would consume greater quantities of these foods.

### Method

#### Participants

Sixty-eight families with children aged between 20 and 24 months were recruited from the University of Reading's Child Development Group database. One child failed to meet the inclusion criteria (see below) and seven families withdrew from the study due to ill health or inability to visit the University for testing. Sixty infants (35 males and 25 females) completed the study, with a mean age of 22 months and 9 days (range 20 months 26 days to 24 months 0 days). Data from three participants (one in each of the liked, disliked and unfamiliar initial status conditions) were excluded from analyses because parents contravened instructions during the test session (*N* = 2) or because the parent changed her report about the initial status of the target vegetable (*N* = 1). Demographic information was provided by all parents; 88% of families were white and 78% came from a household where at least one parent was educated to graduate level. Mothers brought their children to the University for the test session in all but three cases, when other close relatives brought the child. Travel expenses were provided and children were given a certificate and a T-shirt if it was their third visit to the University to participate in a study.

#### Materials

A short-form parent-report “Vegetable Liking and Familiarity Questionnaire” (VLFQ) was created to assess children's familiarity with and liking of 16 different vegetables. These included the five most liked, disliked and unfamiliar vegetables on the original FFLQ (see Experiment 1), after foods that were deemed unsuitable for the taste test were excluded. The VLFQ additionally included spinach, a food that was reported to be unfamiliar to the majority of children on the FFLQ and liked and disliked equally by the remainder (see Supplementary Material for the list of vegetables included). For each vegetable listed, parents were asked to indicate whether the food was liked, disliked or unfamiliar to their child.

A picture-book was produced for each of the vegetables on the VLFQ, with the same layout and format as for Experiment 1 (see Supplementary Material for an example).

Four foods were prepared for each child's taste test, two for the Parent Report Check (one liked, one disliked) and two for the Exposure test (the target food and control food). In addition, two portions of a different vegetable were prepared for the parent to consume, so that the child would not be eating alone (see Houston-Price et al., 2009b). A small portion of each food (equivalent to one teaspoonful) was prepared, so that children could consume any or all of the foods offered without their appetite being affected. Vegetables were washed and presented either raw or cooked, and either sliced or whole, as appropriate. All vegetables were prepared within an hour of the test session and were served at room temperature. To prevent disliked foods “contaminating” liked foods by touching them (Brown and Harris, 2012), each vegetable was served on a separate plate.

#### Procedure

Parents were contacted by telephone and given a brief overview of the experiment. If a parent gave consent to their participation, the child was randomly allocated to one of three initial status conditions (liked, disliked, or unfamiliar), with equal numbers in each condition. The experimenter then verbally administered the VLFQ, asking parents whether their child liked, disliked or had not tried each vegetable listed. For each child, two vegetables were randomly selected from those for which the parent's responses matched the initial status set to which the child had been assigned; these became the target (exposed) and control (non-exposed) foods for that child. Children who were reported to have fewer than two vegetables in the assigned category were excluded from the study (*N* = 16). Parents were then asked to identify a liked and disliked vegetable by means of the following question, “Of all the vegetables you can think of, is there one that you know your child enjoys eating and one that you know they do not enjoy?” The two foods identified by parents were used for that child's Parent Report Check. If the parent named a food that had been selected as the target or control food for the Exposure test, a replacement target or control food was randomly assigned from the foods remaining of that status. Finally, parents were asked whether they would be happy to eat a piece of cucumber (and, if not, some spinach, red pepper, lettuce, or green beans) during the taste test, to help their child feel comfortable about eating in the test environment.

Parents were sent a picture book about their child's target vegetable in the post and were asked to read this with their child for approximately 5 min a day every day for 2 weeks, exactly as in Experiment 1. As before, if parents rescheduled their test date due to unforeseen circumstances such as ill health, they were asked to provide only 14 readings before the rescheduled visit. If the book had been read 14 times before the need to reschedule occurred (*N* = 2), parents were asked to carry out three refresher readings on the 3 days prior to their visit.

Parents brought their child to the University to take part in the taste test at a time when the child was alert and likely to be prepared to eat, typically mid- or late morning or mid- or late afternoon. On arrival at the University, parents were asked to complete the consent form while the researcher played with the child. Parents and children were then taken to the food tasting lab. The child was seated at a low table, with the parent and experimenter seated close by. The parent was reminded about the vegetable that they had agreed to taste and instructed that they should select that vegetable from the tray and should not point to, touch or encourage the child to eat any of the other foods on the tray, other than to repeat requests made by the experimenter. If the child offered the parent a food to eat, the parent was asked to replace it on the child's plate.

Each child took part in the Parent Report Check followed by the Exposure Test. For the Parent Report Check, the researcher brought in a tray containing three small plates; these held the liked and disliked vegetables, as reported by parents, and the food that the parent had agreed to eat. The researcher offered the tray to the parent and parents took the plate containing their designated food. The two plates containing the child's liked and disliked vegetables were then placed in front of the child in fixed side-by-side locations indicated by marked circles on the table. The researcher said to the child, “Here are two vegetables. Which would you like to eat?” If the child tasted a food, the researcher invited them to try the other food. If the child refused to try either vegetable, the researcher named each food and again asked the child which food they would like to try. Children were invited to try each vegetable up to three times. After a period of 5 min (or earlier if the child had eaten both foods or refused to eat any more), the plates were removed.

The Exposure Test followed exactly the same procedure. The parent was offered a tray containing the parent's vegetable and child's target and control vegetables. The child's target and control vegetables were placed on the table and offered to the child as described above. Side of food presentation was fully counterbalanced within trials (so that the liked and disliked and target and control vegetables were placed equally often on the left and right sides) and across trials (so that the target vegetable was placed equally often on the side on which the liked and disliked vegetable had been placed in the previous trial). The test session was coded on-line by the researcher and video-recorded for the purposes of second-coding.

#### Coding

The experimenter recorded children's behavior toward the two foods during each test trial. “Willingness to taste” was coded in terms of three behaviors: (i) whether each food was tasted; (ii) the order in which foods were tasted; and (iii) the encouragement required to persuade the child to taste each food. Tasting was coded when the child placed the food on their lips or tongue, whether the food was subsequently spat out or swallowed. The encouragement required to persuade the child to taste the food was rated on a 5-point scale (1 = very easy to persuade child; 2 = quite easy; 3 = OK; 4 = quite difficult; 5 = very difficult, could not persuade child). “Amount consumed” was coded as a proportion of the portion provided, again using a 5-point scale (0 = none, 1 = nibble, 2 = less than ½tsp, 3 = ½tsp, 4 = whole portion).

As the experimenter was not blind to the liked/disliked or target/control food on each trial (due to the need to counterbalance the side of food presentation), a second blind coder independently coded 20% of the recorded test sessions (*N* = 12). This second coder noted that, while the video footage provided a good view of which foods were tasted, the camera angle and image resolution made it difficult to make fine-grained assessments of the encouragement required to persuade the child to taste the food and the amount of food consumed. The second coder therefore used only a 3-point scale to rate these behaviors (Encouragement required: 1 = easy to persuade, 2 = OK, 3 = difficult; Amount consumed: 1 = none, 2 = some, 3 = all) and the first coder's ratings were collapsed onto the same 3-point scale for reliability checks. Cohen's Kappa statistics for inter-rater reliability ranged from 0.72 to 1.00, representing a high level of agreement. To benefit from the more sensitive coding scheme used by the first coder, the first coder's ratings were used in analyses.

### Results and discussion

According to the reading records provided by parents, children saw their book an average of 14.9 times (*SD* = 9.9) during the exposure phase but, as in Experiment 1, the number of readings varied widely between participants. No child received fewer than 9 readings but one child asked for the book to be read multiple times each day and accrued 84 presentations. As there were no correlations between the number of readings and the continuous measures collected in this study, and as this child was not an outlier on any measure, no participant was excluded from analyses on the basis of the number of readings experienced.

#### Parent report check

Table 2 presents the results of the parent report check trial. Of the 57 participants, 21 children tasted both the “liked” and “disliked” vegetables, 24 tasted only the liked food, 4 tasted only the disliked food, and 8 tasted neither. A chi-square test showed that there was a significant association between whether children tasted a food and whether it was reported to be liked or disliked by the parent, χ^2^_(1)_ = 15.41, *p* < 0.001; more children tasted the food reported to be liked. Of the 49 children who tasted at least one food on this trial, 38 tasted the liked vegetable first and 11 tasted the disliked vegetable first. A binomial test confirmed that significantly more children tasted the liked vegetable first (*N* = 49, *p* < 0.001).

**Table 2:** **Number of children who tasted the foods reported by parents to be “liked” and “disliked,” the number who tasted each of these foods first, and mean ratings of the degree of encouragement required to persuade the child to taste each food (1 = very easy, 5 = very difficult) and amount of each food consumed (0 = none, 4 = whole portion)**.

**Food**	***N* who tasted this food**	***N* who tasted this food first**	**Encouragement required Mean (*SD*)**	**Amount consumed Mean (*SD*)**
“Liked”	45	38	2.3 (1.7)	2.5 (1.7)
“Disliked”	25	11	4.3 (1.4)	0.5 (1.1)

We also compared the encouragement required to persuade children to taste the foods reported to be liked and disliked (see Table 2). A Wilcoxon signed ranks test confirmed that significantly less encouragement was required to persuade children to taste the vegetable that was reported to be liked (*Z* = −5.15, *p* < 0.001). Finally, there was a significant difference in the amount of the two foods consumed; children ate more of the liked vegetable than the disliked vegetable (*Z* = −5.03, *p* < 0.001).

These analyses show that, in line with the findings of Linneman et al. (2004), parents of young children can accurately report on the vegetables their children like and dislike. Compared to the food reported to be disliked, the food that was reported to be liked was tasted by more children, tasted first by more children, required less encouragement to be eaten and was consumed in greater quantities.

#### Exposure test

Table 3 presents the results of the exposure test trial for the children in each initial status condition separately, and for all children combined. Of the 57 children who took part, 30 tasted both the target and control foods, 13 tasted only the target vegetable, 6 tasted only the control vegetable, and 8 tasted neither. A chi-squared test found no association between whether a vegetable had been exposed or not and whether it was tasted in the test trial, χ^2^_(1)_ = 3.29, *p* = 0.07. We explored whether this pattern was true for each of the three initial status groups using a 2 (target vs. control) × 3 (initial status category) × 2 (whether the food was tasted) log-linear analysis. This found no main effect of exposure, *G*^2^_(1)_ = 2.04, *p* = 0.15, no main effect of initial status condition, *G*^2^_(2)_ = 2.04, *p* = 0.36, and no interaction between exposure and initial status category, *G*^2^_(7)_ = 4.14, *p* = 0.76. Thus, whether children tasted a food or not was not influenced by whether it had been seen in their picture book or its initial status as liked, disliked or unfamiliar.

**Table 3:** **Number of children who tasted the target and control foods, the number who tasted each of these first, and mean ratings of the degree of encouragement required to persuade the child to taste each food (1 = very easy, 5 = very difficult) and amount of each food consumed (0 = none, 4 = whole portion), for each initial status condition and for all groups combined**.

**Initial status condition**	***N***	**Food**	***N* who tasted this food**	***N* who tasted this food first**	**Encouragement required Mean (*SD*)**	**Amount consumed Mean (*SD*)**
Liked	19	Target	15	9	2.5 (1.8)	2.2 (1.7)
		Control	13	7	3.0 (1.9)	1.9 (1.8)
Disliked	19	Target	13	9	3.9 (1.5)	0.7 (1.0)
		Control	10	7	4.2 (1.4)	0.5 (1.0)
Unfamiliar	19	Target	15	12	2.4 (1.8)	2.0 (1.7)
		Control	13	5	3.7 (1.7)	1.0 (1.6)
All	57	Target	43	30	2.9 (1.8)	1.6 (1.6)
		Control	36	19	3.6 (1.7)	1.1 (1.6)

The order in which children tasted the target and control vegetables was also examined (see Table 3). Of the 49 children who tasted at least one food, 30 tasted the target vegetable first and 19 tasted the control vegetable first, a distribution that was not different to chance in a binomial test (*N* = 49, *p* = 0.15). The same pattern held for each initial status condition; there was no association between a food's initial status as liked, disliked or unfamiliar and whether the target or control food was tasted first, χ^2^_(2)_ = 0.02, *p* = 0.66. Children were equally likely to select the vegetable that they had seen in their picture books and the control vegetable to taste first.

The next set of analyses explores the encouragement required to persuade children to eat the target and control foods (see Table 3). A Wilcoxon signed ranks test showed that significantly more encouragement was required to persuade children to taste the control vegetables than the target vegetables, *Z* = −3.14, *p* = 0.001. When the groups were split by initial status condition, children who were exposed to unfamiliar vegetables required more encouragement to taste the control vegetable than the target vegetable, *Z* = −2.69, *p* = 0.007. No significant differences was seen between the degree of encouragement required to persuade children in the liked (*Z* = −1.32, *p* = 0.19) or disliked (*Z* = −1.38, *p* = 0.17) conditions to taste the target and control vegetables, although the pattern was similar across the three groups. Thus, the experimenter found it easier to encourage children to taste the vegetable that they had seen in their picture-books, especially when children had not tried either food before.

Finally, we examined the amount of each food consumed by children (see Table 3). Overall, children consumed more of the target vegetable than of the control vegetable, *Z* = −2.4, *p* = 0.016. Again, while the pattern was broadly similar across the three groups, it was only the children in the unfamiliar initial status condition who consumed significantly more of the target vegetable (liked: *Z* = −0.77, *p* = 0.44; disliked: *Z* = −0.95; *p* = 0.34; unfamiliar: *Z* = −2.5, *p* = 0.011). The picture books therefore increased the amount of the target food consumed, particularly where the vegetable was unfamiliar at the start of the intervention.

To summarize, the results of the Parent Report Check confirmed that parents are able to report accurately on their child's likes and dislikes in relation to vegetables. Children were more willing to taste the vegetable that they were reported to like (as evidenced by the number of children who tasted the liked vs. disliked foods, the order in which these were tasted, and the level of encouragement required to persuade the child to eat them) and consumed more of the vegetable that they were reported to like.

The results of the Exposure Test were less systematic. First, whether a vegetable had been seen in a child's picture book did not influence whether it was tasted. This is perhaps not surprising given that children were repeatedly encouraged to taste both foods, but there was also no effect of exposure on the order in which children tasted the two foods. On their own, these findings suggest that the books did not affect children's willingness to taste the foods in them. Houston-Price et al. (2009b) similarly found no positive effects of exposure on either the frequency with which children tasted vegetables or the order in which they tasted them; in their study, the positive results pertained only to fruits. In both studies, the books' effects were sought in differences in children's behavior toward the target and control foods. While we expected control foods to provide a “baseline” measure of the child's willingness to consume a food of the same initial status as the target food, it is possible that reading the book could have affected the child's willingness to try other vegetables, in addition to the food targeted. This would, of course, have confounded the detection of differences in children's behavior toward the target and control foods at test. To explore this possibility, future studies should include a control group of children who do not see a book prior to testing, against whom the experimental group's eating behavior can be compared.

However, in contrast to our previous study, Experiment 2 found no negative effects of exposure to vegetables and, importantly, the additional measures collected in this study revealed some positive effects. Experimenter ratings indicated that less effort was needed to persuade children to taste target vegetables than control vegetables, particularly for foods that were unfamiliar to children prior to the study. The parallel behavior shown toward reportedly liked foods in the Parent Report Check gives us confidence in interpreting children's behavior toward target foods in terms of a greater willingness to taste these. The same pattern was seen in children's consumption of the target and control foods: children ate more of the target vegetable than of the control vegetable, and again this was particularly the case for foods that children had not tried previously. This was a rather surprising finding; while we had hypothesized that familiarity with the appearance of a food would increase children's willingness to try it, there was no reason to expect visual familiarity to enhance their liking of the food's taste. On the other hand, one might expect a similar pattern to be seen in measures of willingness to taste and amount consumed in this type of study, because a child who is willing to taste a food several times will necessarily eat more of it. If levels of food consumption are taken as an indication of food acceptance and food liking (Cooke and Wardle, 2005), this study provides the first evidence that picture books can be used to increase young children's vegetable intake. Moreover, the fact that infants ate more of the target vegetable on the very first occasion the food was offered suggests that picture books might eliminate the need for repeated taste exposures when parents are attempting to introduce new vegetables into their child's diet.

## General discussion

The experiments reported here corroborate previous reports that looking at picture books about fruits and vegetables increases infants' interest in looking at these foods (Experiment 1) and additionally demonstrate that such books can reduce the encouragement a child requires to taste a food and increase the amount of the food they consume (Experiment 2). Both studies confirmed that the impact of fruit and vegetable books depends upon the status of the food depicted, with the most positive effects seen for foods that were initially unfamiliar. Importantly, neither study found any negative effects of looking at books about foods, allaying fears raised by previous research that children might be less likely to taste familiar foods if these were repeatedly seen in picture books (Houston-Price et al., 2009b).

Houston-Price et al. (2009b) similarly reported more positive effects for foods that were initially unfamiliar to children. Fruit and vegetable books may therefore be most useful when a child is first introduced to a new food. Previous studies have shown that parents often fail to provide their child with sufficient taste exposures to a new food to bring about acceptance, due to the “bothersome behavior” children display when faced with new foods (Carruth and Skinner, 2000; Carruth et al., 2004). The current studies suggest that children might be more easily persuaded to try a new food and more accepting of its taste if they look at a book about the food before it is offered. Assuming that parents find repeatedly looking at a picture book about a food less stressful than repeatedly offering their child the food to eat, our findings suggest that picture books might help parents bypass some of the difficulties associated with introducing new vegetables.

While our two studies concurred in finding the strongest effects of exposure for initially unfamiliar foods, the visual preference data collected in Experiment 1 revealed a more graded effect of food status; the books had the least impact when they depicted foods that were already liked by the child and a moderate effect when disliked foods were shown. We are confident in interpreting these results in terms of genuine differences in the effectiveness of visual exposure to foods of differing initial status, for two reasons. First, children's behavior in the Parent Report Check of Experiment 2 unequivocally confirmed parents' ability to accurately report whether a vegetable was liked or disliked by their child. Across all measures, children were more willing to taste a vegetable that was reported to be “liked,” and consumed more of this food, than of a vegetable that was reported to be “disliked.” Second, in both experiments, the very existence of differences in children's behavior toward the foods in the differing initial status conditions confirms that the foods in each condition belonged to different categories for the child. That is, if parents had been unable to appropriately categorize foods as liked, disliked or unfamiliar, we would have seen no differences in children's behavior toward the foods in the different initial status conditions. In contrast, in both studies, children were affected differentially by the intervention depending on the food status condition to which they had been assigned. In relation to this point, it is worth noting that the failure to find an effect of exposure for liked foods in Experiment 2 was largely a consequence of children's willingness to consume both the target and control foods in this condition (see Table 3).

Interesting questions remain about the mechanisms by which picture books enhance children's interest in looking at and tasting the subject matter. As this type of intervention relies upon children spending time looking at the featured food, visual familiarity with the food is very likely to be central to its success. “Mere exposure” effects—whereby even very brief exposures to a stimulus can enhance participants' reports of how much they like the stimulus—have been demonstrated for a variety of types of visual stimuli, ranging from abstract shapes, such as Chinese characters (Monahan et al., 2000), to meaningful social stimuli, such as human faces (Zajonc, 1968, 2001). According to the “perceptual fluency” account of the mere exposure phenomenon, participants' positive attitudes toward exposed stimuli are attributable to the greater ease with which perceptual systems process stimuli that have previously been encountered (Bornstein and D'Agostino, 1994). The discovery of stronger exposure effects among the children who were shown exactly the same pictures of target foods that they had seen in their books in Experiment 1 suggests that perceptual fluency is likely to have been a factor in this study. By this view, the stronger exposure effects shown by children in the unfamiliar initial status condition would be due to the particular effort associated with forming a perceptual representation of the completely unfamiliar control foods, relative to the newly-familiarized target foods.

It is less clear, however, that perceptual fluency can account for children's behavior toward the target foods in Experiment 2, as these would have appeared perceptually quite different to the pictures children saw in their books. An interesting, alternative possibility is that the influence of the picture books arises through the foods' “learned safety” (Kalat and Rozin, 1973). The positive effects of repeated taste exposure on food liking (Birch and Marlin, 1982; Birch et al., 1987; Sullivan and Birch, 1990; Wardle et al., 2003a,b; Lakkakula et al., 2010) are often attributed to the child learning that a food is safe to eat as a result of a lack of negative consequences of consuming it. Zajonc (2001) argues that a similar mechanism accounts for mere exposure effects in other domains; repeated exposure to any stimulus without aversive consequence conditions us to learn that the stimulus is safe to approach. The implication of this claim is that our learning mechanisms do not distinguish between real world stimuli, which vary in how safe they are to approach, and pictorial stimuli, which do not. Children's greater willingness to taste the foods to which they had been visually exposed in Experiment 2 would, by this account, reflect the learned safety that resulted from exposure to pictures of the foods. The stronger exposure effect seen for unfamiliar foods would be explained in terms of children's complete uncertainty about the safety of the unfamiliar control food in this condition, in contrast to the control foods offered to children in the liked and disliked conditions, which would have been tasted, and discovered to be safe, before.

Visual familiarity is not the only factor likely to have contributed to the impact of the intervention on children's behaviors toward targeted foods, of course. Children could also have learned about foods through the verbal descriptions provided in the books, which included both neutral and positive statements. Children are thought to organize their knowledge about foods in schemas, stored bodies of knowledge that facilitate the rapid processing and interpretation of information and determine how we respond to stimuli in future (Fiske and Taylor, 1991; Pliner, 2008; Vereijken et al., 2011). The changes we observed in children's behavior toward the target foods could reflect the assimilation of the positive statements children heard about the tastiness of the target food (e.g., “Carrots are great to eat raw because they are crunchy”) into their schema for the food. The fact that picture books were most effective for unfamiliar foods, for which children would have held no pre-existing schema, suggests that it might be more challenging to adjust a pre-existing schema than to construct a positive schema for a new food from the outset. For liked foods, for which positive schemas are already held, there may be little scope for the information in picture books to enhance the status of the food. For disliked foods, the positive messages may be insufficient to overcome the child's stored memories of negative experiences with the food.

A further factor that might have contributed to the effects of the books is the manner in which they were delivered. Just as favorable experiences with foods lead to the development of positive schemas and expectations of liking of them (Pliner, 2008), the presumably enjoyable shared reading sessions with parents could support the development of positive expectations about the contents of the book. Anecdotal reports from the families who participated in these studies revealed that some parents attempted to make the book more enjoyable for their child by pretending to chop or eat the food shown. Previous research has shown that interactive reading styles are optimal for preschoolers' learning of the vocabulary contained in story books (see Mol et al., 2001, for a review); it is likely that such benefits extend to the learning of other aspects of a book's content. However, the nature of the interaction is likely to matter. One parent admitted adding the word “Yuk!” after reading every page of her child's book about mushrooms; unsurprisingly, the book did not have a positive effect for this child. Should picture books be recommended to parents as a way of introducing their toddler to a new food, we would encourage the parents who take up the opportunity to approach the process with a positive attitude.

It is important to highlight the role played by parents during the intervention, not only as readers of the book, but also as the providers of food for the family during the reading period. It is quite possible that the effects of our manipulation were driven by changes to the parents' attitudes and behaviors in relation to the target food, rather than, or in addition to, changes within the children. For example, reading a book about a little-known vegetable might increase the parent's interest in the food described and lead them to purchase or provide the food more frequently within the home. Although we have not tested this hypothesis, it is supported by anecdotal evidence from the families in our studies: one mother reported planting carrot seeds with her child after reading his book about carrots; another sent us photographs of a trip she and her child had made to a broccoli farm after reading a book about broccoli. Several parents reported that they had pointed out or bought their child's target food whilst in the supermarket. A more detailed investigation of the parental behaviors that accompany the sharing of picture books with children would help to establish the extent to which the positive outcomes of the intervention are a direct result of the books' influence on children's willingness to engage with the targeted foods vs. an indirect consequence of the books' influence on their parents.

There is therefore certainly more to learn about how picture books influence children's behaviors toward foods. Further work is also needed to establish how a picture-book intervention might be optimized. In neither the current studies nor in our previous work (Houston-Price et al., 2009a) have we found the number of exposures children receive to their book to determine the strength of the exposure effect; future studies should therefore seek to ascertain the minimum number of exposures required for a positive outcome. Work is also needed to optimize the content and style of the books for young children. The findings of previous research would suggest that the use of photographic images of foods should facilitate children in transferring what they learn from books to the foods they are offered at home (Ganea et al., 2008). However, some have found cartoon story books to have positive effects on eating behavior (de Droog et al., 2014) and, as yet, no study has directly compared these to the photo-style books used in the current studies. The books might also benefit from the inclusion of pictures of peers eating the target foods. Peer models are known to influence young children's eating habits (Birch, 1980) and are a key component of interventions to increase children's fruit and vegetable intake in nurseries and pre-schools (Lowe and Horne, 2009; Horne et al., 2011).

Our findings also hint at interesting new avenues for research into picture books, particularly their potential to bring about positive attitudes toward non-food stimuli. For example, book exposure might be used to familiarize children with creatures such as insects or spiders, which often promote unnecessary anxiety. Picture books might also be used for public health purposes; reading a toddler a book about teeth cleaning might increase the child's willingness to have their teeth cleaned. Effects might also translate to social stimuli; prior to a visit to an unfamiliar relative, a parent might show a child photographs of the person, to help the child feel comfortable about spending time with them. Thus, in addition to cultivating an enthusiasm for vegetables, picture books could prove to be a useful tool for supporting development more generally.

### Conflict of interest statement

The authors declare that the research was conducted in the absence of any commercial or financial relationships that could be construed as a potential conflict of interest.
