# Supplementary material for: Let's look at leeks! Picture books increase toddlers' willingness to look at, taste and consume unfamiliar vegetables
Source: Front Psychol. 2014 Mar 11;5:191. doi: 10.3389/fpsyg.2014.00191 (PMC3949128; doi:10.3389/fpsyg.2014.00191)
Supplement: Supplementary file 1 [file Presentation1.PDF]

Supplementary Material

Appendix i

The six fruits and vegetables selected for each of the initial status sets in Experiment 1

Liked Fruit:
Apple, banana, clementine, grape, pear, raisin
Liked Vegetables:
Broccoli, carrot, onion, peas, sweet corn, sweet potato
Disliked Vegetables:
Avocado, Cabbage, green beans, lettuce, mushroom, tomato
Unfamiliar Fruit
Gooseberry, guava, lychee, passion fruit, pomegranate, redcurrant
Unfamiliar Vegetables:
Asparagus, beetroot, marrow, radish, turnip, water cress

Appendix ii

Example of a book used in the ‘Liked Vegetable’ condition of Experiment 1.

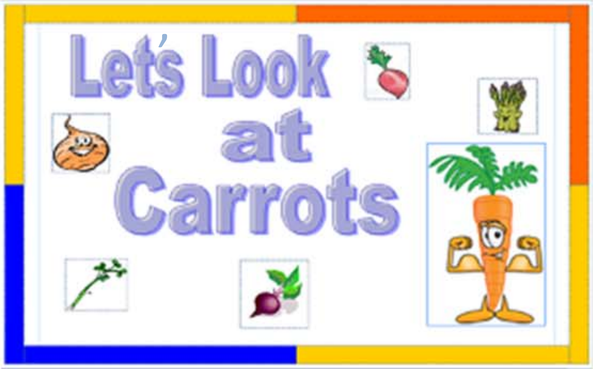

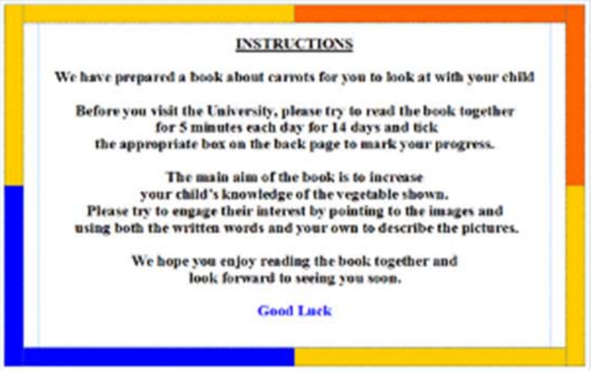

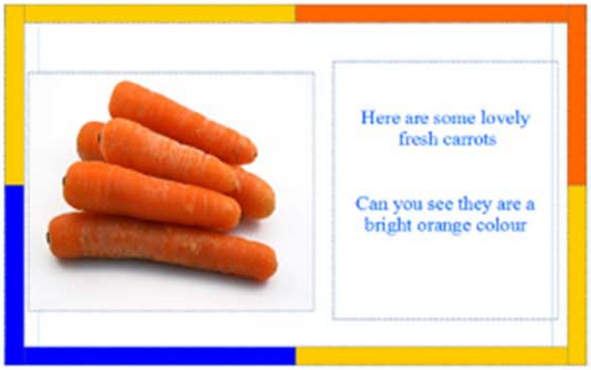

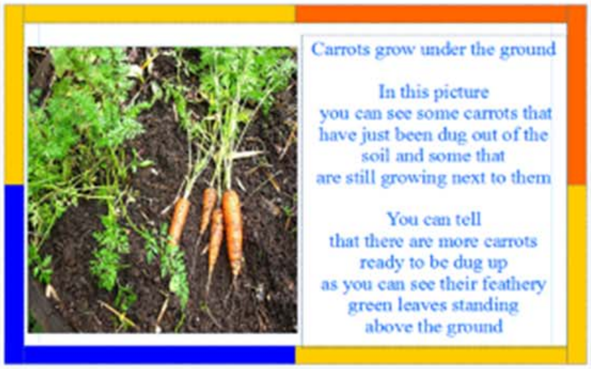

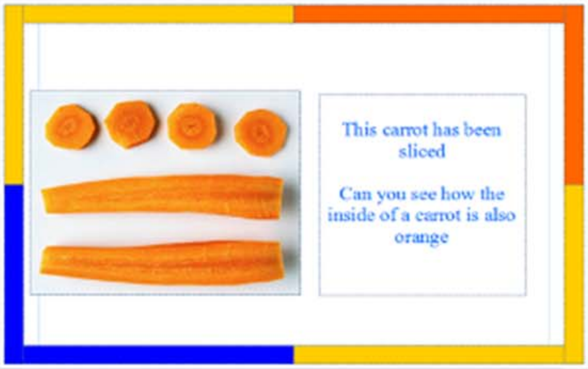

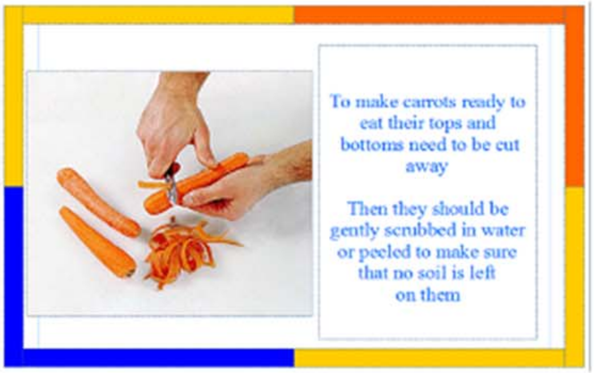

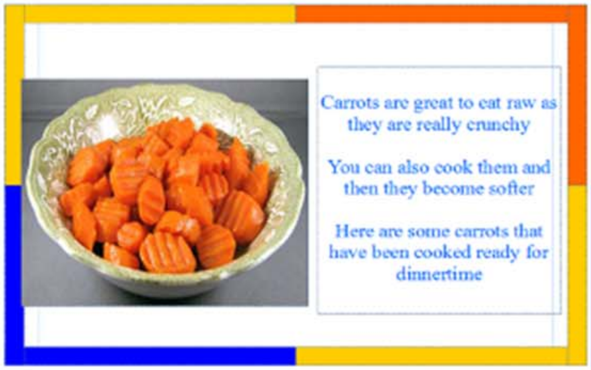

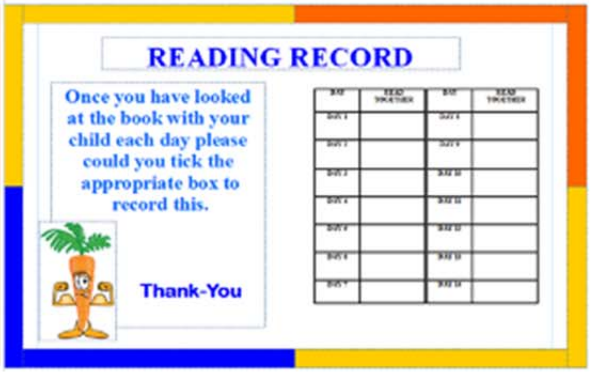

Appendix iii

The following 16 vegetables were on the list provided to parents in Experiment 2. Depending on each child's familiarity with and liking of the foods, a food might be selected for any of the initial status conditions:

Asparagus, avacado, broccoli, cabbage, carrots, celery, green beans, lettuce, peas, radish, red pepper, spinach, spring onions, sweet corn, tomato, watercress
